# Correction: Four new species of *Pristimantis* Jiménez de la Espada, 1870 (Anura: Craugastoridae) in the eastern Amazon

**DOI:** 10.1371/journal.pone.0243182

**Published:** 2020-11-25

**Authors:** Elciomar Araújo de Oliveira, Leandro Alves da Silva, Elvis Almeida Pereira Silva, Karen Larissa Auzier Guimarães, Marcos Penhacek, José Gregório Martínez, Luís Reginaldo Ribeiro Rodrigues, Diego José Santana, Emil José Hernández-Ruz

There is an error in the caption for Fig 5. Please see the complete, correct Fig 5 caption here.

**Fig 5:**
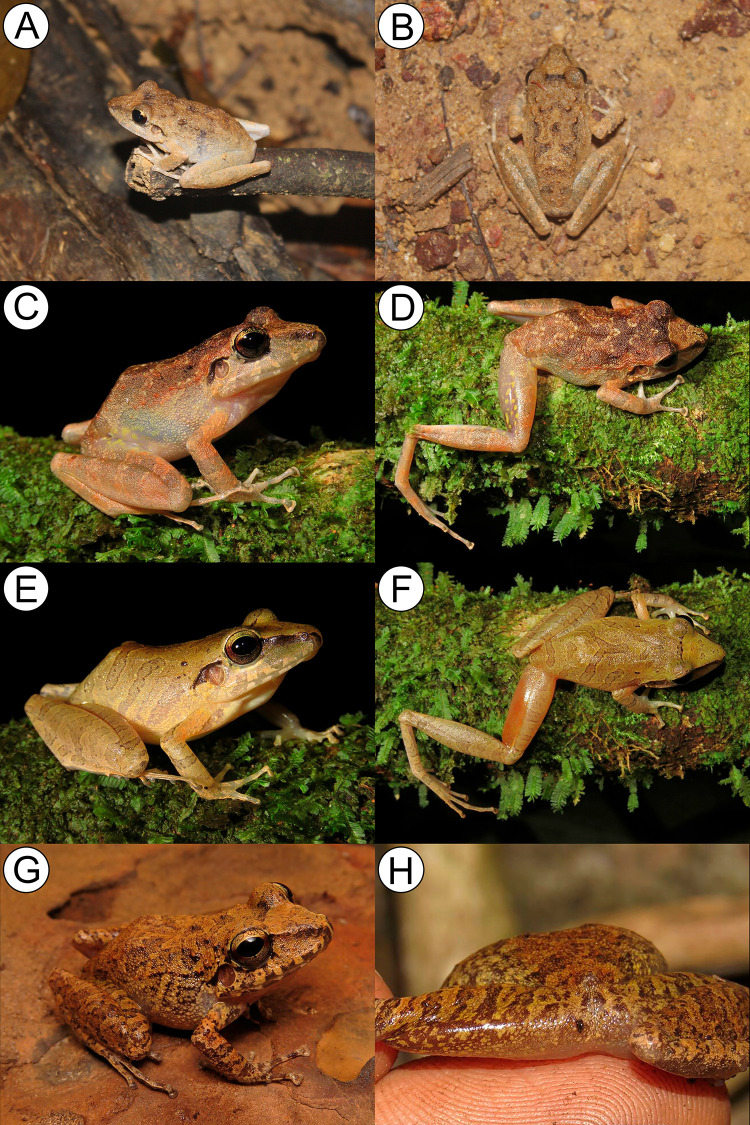
Color in life of: *Pristimantis giorgii* sp. nov. Pará state (A—B); *P*. *pictus* **sp. nov.** Mato Grosso state (C–D); *P*. *pluvian* **sp. nov.** Mato Grosso state (E–F) and *P*. *moa* **sp. nov.** Tocantins state (G–H).

The image for Fig 6 is incorrect. Please see the complete, correct Fig 6 here.

**Fig 6:**
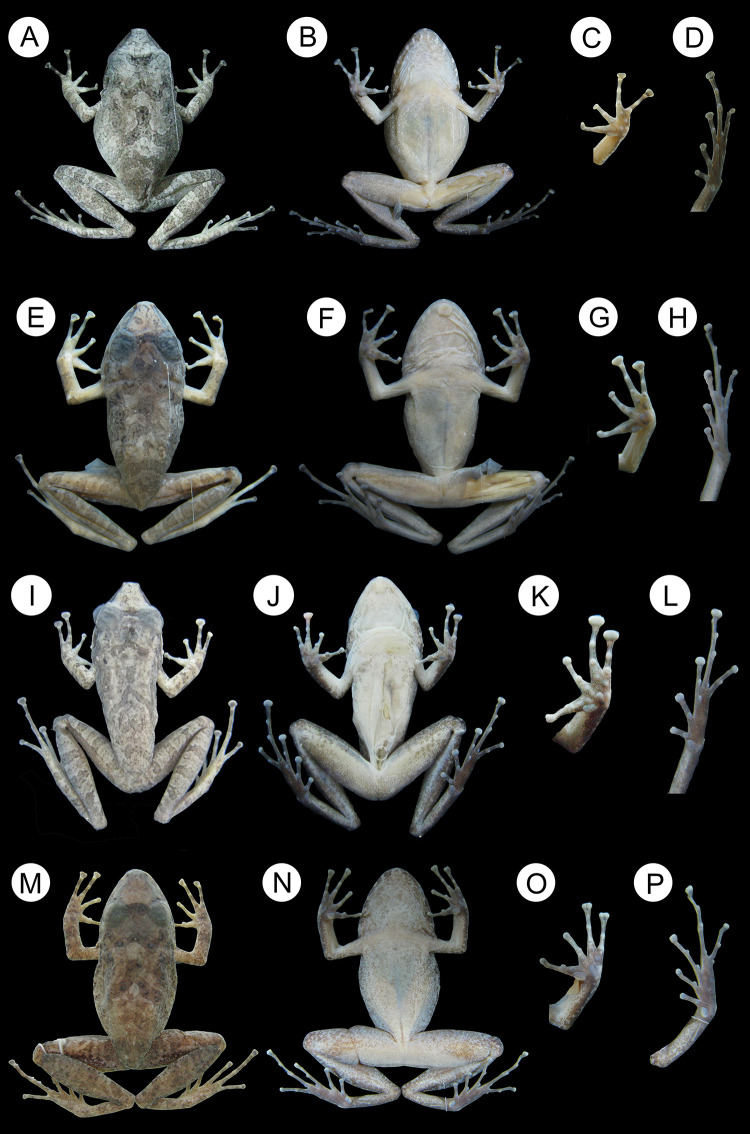
Holotype of *Pristimantis giorgii* sp. nov. (Portel, State of Pará): A) dorsal view (SVL = 34.2 mm), B) ventral view; C) left hand (8.9 mm) and D) left foot (16 mm). Holotype of *Pristimantis pictus* **sp. nov.** (Novo Mundo, State of Mato Grosso): E) dorsal view (SVL = 30.6 mm), F) ventral view; G) left hand (8.2 mm) and H) left foot (15.8 mm). Holotype of *Pristimantis pluvian* **sp. nov.** (Cotriguaçu, State of Mato Grosso): I) dorsal view (SVL = 28.7 mm), J) ventral view; K) right hand (7.3 mm) and L) right foot (14 mm). Holotype of *Pristimantis moa* **sp. nov.** (Palmeirante, Tocantins State): M) dorsal view (SVL = 32.9 mm), N) ventral view; O) left hand (8.8 mm) and P) right foot (15.9 mm).
