# Supplementary material for: Four new species of Pristimantis Jiménez de la Espada, 1870 (Anura: Craugastoridae) in the eastern Amazon
Source: PLoS One. 2020 Mar 18;15(3):e0229971. doi: 10.1371/journal.pone.0229971 (PMC7080247; doi:10.1371/journal.pone.0229971)
Supplement: S1 File — (PDF) [file pone.0229971.s001.pdf]

PCR Details

16S

Amplification was performed under the following conditions: 60s at 92°C followed by 35 cycles of 92°C (60 s), 50°C (50 s) and 72°C (1.5 min). The final volume of the PCR reaction was 12 µL and it contained 4.4 µL of ddH_2O, 1.5 µL of 25 mM $MgCl_2$, 1.25 µL of 10 mM dNTPs (2.5mM each dNTP), 1.25 µL of buffer 10x (75 mM Tris HCl, 50 mM KCl, 20 mM $(NH_4)_2SO_4$), 1 µL of each *primer* (2 µM), 0.3 µL of 1 U Taq DNA Polymerase and 1 µL of DNA (30 – 50 ng/µL).

COI

Amplification was performed under the following conditions: 3 min at 95 °C, followed by 35 cycles from 30s to 95°C, 30s to 53 °C, 40s to 72 °C and a final extension of 7 min. to 72 °C. The final volume of the PCR reaction was 15 µL and it contained 7.1 µL ddH_2O, 1.6 µL de 25 mM $MgCl_2$, 1.25 µL of 10 mM dNTPs (2.5mM each dNTP), 1.25 µL of buffer 10x (75 mM Tris HCl, 50 mM KCl, 20 mM $(NH_4)_2SO_4$), 1.5 µL of *primer* LCO 1490 (5'-GGTCAACAAATCATAAAGATATTG-3') and HCO 2198 (5'-TAAACTTCAGGGTGACCAAAAAATCA-3') (Folmer *et al.,* 1994), 0.3 µL of 1 U Taq DNA Polymerase and 1 µL of DNA (30 – 50 ng/µ).

Sequencing reaction Protocol

Sequencing reactions were made in 96-well plates with final volume of 10µL, containing 4 µL of sterile H_2O, 1.5 µL of sequencing buffer 5X, 0.5 µL of primer (10 µM), 1 µL of Big Dye mixture and 3 µL of PCR cleaned product. PCR conditions were as follows: 96°C (1 min); 35 cycles of 96°C (15 sec), 50°C (15 sec), and 60°C (4 min). The reactions were precipitated in ethanol/EDTA and dried at 90°C for 2 min. The plates were re-suspended with 10 µL Formamide Hi-Di, heated at 94°C for 3 min. and sequenced in ABI 3500xl genetic analyzer (Applied Biosystems™).
