# Supplementary material for: Four new species of Pristimantis Jiménez de la Espada, 1870 (Anura: Craugastoridae) in the eastern Amazon
Source: PLoS One. 2020 Mar 18;15(3):e0229971. doi: 10.1371/journal.pone.0229971 (PMC7080247; doi:10.1371/journal.pone.0229971)
Supplement: S2 File — (PDF) [file pone.0229971.s002.pdf]

List of specimens used for molecular analysis. MT = Mato Grosso; PA = Pará; TO = Tocantins; MA = Maranhão; AM = Amazonas

Species	Localities	GenBank 16S/COI	Nº in colection	Status of specimens	Source
P. pictus	Cotriguaçu, MT	MK992501/-	ABAM3077	Voucher	This study
P. pictus	Cotriguaçu, MT	MK992502/MN010653	ABAM3078	Voucher	This study
P. pictus	Cotriguaçu, MT	MK992502/-	ABAM3081	Voucher	This study
P. pictus	Novo Mundo, MT	MK992504/MN010654	ABAM2154	Voucher	This study
P. pictus	Novo Mundo, MT	MK992505/MN010655	ABAM1525	Voucher	This study
P. pictus	Novo Mundo, MT	MK992506/MN010656	ABAM1829	Voucher	This study
P. pictus	Novo Mundo, MT	MK992507/MN010657	ABAM1831	Voucher	This study
P. pictus	Novo Mundo, MT	MK992508/MN010658	ABAM2129	Voucher	This study
P. pictus	Novo Mundo, MT	MK992509/MN010659	ABAM2150	Voucher	This study
P. pictus	Novo Mundo, MT	MK992510/MN010660	ABAM2161	Voucher	This study
P. pictus	Novo Mundo, MT	MK992511/-	ABAM2151	Voucher	This study
P. pictus	Jacareacanga, PA	MK992512/MN010661	LA53		This study
P. pictus	Jacareacanga, PA	MK992513/-	LA51	Voucher	This study
P. pictus	Paranaíta, MT	MK992514/-	MAP1423	Voucher	This study
P. pictus	Paranaíta, MT	MK992515/-	MAP1424	Voucher	This study
P. pictus	Paranaíta, MT	MK992516/MN010662	MAP1445	Voucher	This study
P. pictus	Paranaíta, MT	MK992517/MN010663	MAP1435	Voucher	This study
P. pictus	Paranaíta, MT	MK992518/MN010664	MAP1439	Voucher	This study
P. pictus	Paranaíta, MT	MK992519/MN010665	MAP1443	Voucher	This study
P. pictus	Jacareacanga, PA	MK992520/MN010666	LA70	Voucher	This study
P. pictus	Jacareacanga, PA	MK992521/MN010667	LA29	Voucher	This study
P. pictus	Cristalino, MT	MK992522/-	ABAM2148	Voucher	This study
P. pictus	Novo Mundo, MT	MK992523/MN010668	ABAM1830	Voucher	This study
P. pictus	Cristalino, MT	MK992524/MN010669	ABAM2146	Voucher	This study
P. pictus	Novo Mundo, MT	-/MN010670	ABAM1602	Voucher	This study
P. moa	Palmas, TO	MK992525/MN010671	MAP0318	Voucher	This study
P. moa	Palmas, TO	MK992526/MN010672	MAP1659	Voucher	This study
P. moa	Palmas, TO	MK992527/MN010673	MAP1660	Voucher	This study
P. moa	Riachão, MA	MK992528/MN010674	LA65	Voucher	This study
P. moa	Riachão, MA	MK992529/MN010675	LA62	Voucher	This study
P. moa	Riachão, MA	MK992530/MN010676	208	Voucher	This study
P. moa	Riachão, MA	MK992531/M	211	Voucher	This study

		N010677			
P. moa	Araguaína, TO	MK992532/MN010678	MAP1728	Voucher	This study
P. moa	Araguaína, TO	MK992533/MN010679	LA24	Voucher	This study
P. moa	Araguaína, TO	MK992534/MN010680	LA34	Voucher	This study
P. moa	Araguaína, TO	KX242518/-	114		This study
P. moa	Palmas, TO	MK992535/MN010681	MAP0319	Voucher	This study
P. moa	Palmas, TO	MK992536/MN010682	MAP0320	Voucher	This study
P. moa	Palmas, TO	MK992537/MN010683	MAP1654	Voucher	This study
P. moa	Palmas, TO	MK992538/MN010684	MAP1655	Voucher	This study
P. moa	Palmas, TO	MK992539/MN010685	MAP1656	Voucher	This study
P. moa	Palmas, TO	MK992540/MN010686	MAP1657	Voucher	This study
P. moa	Palmas, TO	MK992541/MN010687	MAP1658	Voucher	This study
P. moa	Palmas, TO	MK992542/MN010688	MAP1661	Voucher	This study
P. moa	Palmas, TO	MK992543/-	MAP1662	Voucher	This study
P. moa	Palmas, TO	MK992544/-	MAP1663	Voucher	This study
P. moa	Palmas, TO	MK992545/-	MAP1664	Voucher	This study
P. moa	Palmas, TO	MK992546/MN010689	MAP1665	Voucher	This study
P. moa	Palmas, TO	MK992547/MN010690	MAP1666	Voucher	This study
P. moa	Palmas, TO	MK992548/MN010691	MAP2189	Voucher	This study
P. moa	Palmas, TO	MK992549/MN010692	B44	Voucher	This study
P. moa	Palmas, TO	MK992550/-	B45	Voucher	This study
P. moa	Riachão, MA	MK992551/MN010693	207	Voucher	This study
P. moa	Riachão, MA	MK992552/MN010694	209	Voucher	This study
P. moa	Riachão, MA	MK992553/-	210	Voucher	This study
P. moa	Carolina, MA	KT221610/-			This study
P. moa	Araguaína, TO	MK992554/MN010695	LA77	Voucher	This study
P. moa	Araguaína, TO	MK992555/MN010696	D30	Voucher	This study
P. moa	Palmas, TO	-/MN010697	MAP317	Voucher	This study
P. moa	Araguaína, TO	-/MN010698	MAP2669	Voucher	This study
P. moa	Araguaína, TO	-/MN010699	LA111	Voucher	This study
P. giorgii	Assuruni, PA	-/MN010700	SF27	Voucher	This study
P. giorgii	Assurini, PA	MK992556/MN010701	SF21	Voucher	This study
P. giorgii	Assurini, PA	-/MN010702	SF19	Voucher	This study
P. giorgii	Assurini, PA	-/MN010703	SF17	Voucher	This study
P. giorgii	Caxiuanã, PA	MK992557/MN010706	CAX19	Voucher	This study
P. giorgii	Caxiuanã, PA	MK992558/MN010704	CAX35	Voucher	This study
P. giorgii	Caxiuanã, PA	MK992559/MN010705	CAX47	Voucher	This study

P. giorgii	Caxiuanã, PA	MK992560/-	CAX01	Voucher	
P. giorgii	Tucuruí, PA	KX242516/-	MPEG17745	Voucher	De Oliveira et al. 2017
P. giorgii	Marabá, PA	KX242515/KX259216	MPEG 0049	Voucher	De Oliveira et al. 2017
P. giorgii	Marabá, PA	MK992561/MN010707	MPEG 0221	Voucher	De Oliveira et al. 2017
P. giorgii	Marabá, PA	MK992562/MN010708	MA130		This study
P. giorgii	Portel, PA	-/MN010709	FUP56	Voucher	This study
P. giorgii	Portel, PA	MK992563/MN010710	FUP29	Voucher	This study
P. giorgii	Portel, PA	MK992564/MN010711	FUP28	Voucher	This study
P. giorgii	Portel, PA	MK992565/MN010712	FUP52	Voucher	This study
P. giorgii	Portel, PA	MK992566/MN010713	FUP53	Voucher	This study
P. giorgii	Portel, PA	MK992567/MN010714	LZA1365	Voucher	This study
P. giorgii	Assurini, PA	MK992568/-	BLM1141	Voucher	This study
P. giorgii	Assurini, PA	MK992569/-	BLM1142	Voucher	This study
P. giorgii	Marabá, PA	JX267540/-	MZUSP139440	Voucher	This study
P. giorgii	Marabá, PA	KU495457/KU494664/	CFBHT12087	Voucher	This study
P. giorgii	Portel, PA	MK992570/MN010715	FUP32	Voucher	This study
P. giorgii	Portel, PA	MK992571/MN010716	FUP38	Voucher	This study
P. giorgii	Portel, PA	MK992572/MN010717	FUP50	Voucher	This study
P. giorgii	Portel, PA	MK992573/-	FUP54	Voucher	This study
P. pluvian	Cotriguaçu, MT	MK992574/MN010718	ABAM 1556	Voucher	This study
P. pluvian	Cotriguaçu, MT	MK992575/MN010719	ABAM 2173	Voucher	This study
P. pluvian	Cotriguaçu, MT	MK992576/-	ABAM 742	Voucher	This study
P. pluvian	Cotriguaçu, MT	MK992577/MN010720	ABAM 1348	Voucher	This study
P. pluvian	Paranaíta, MT	-/MN010721	MAPT01793		This study
P. pluvian	Paranaíta, MT	-/MN010722	MAP2338	Voucher	This study
P. pluvian	Paranaíta, MT	-/MN010723	MAP2341	Voucher	This study
P. pluvian	Paranaíta, MT	-/MN010724	MAP2353	Voucher	This study
P. pluvian	Paranaíta, MT	-/MN010725	LA52	Voucher	This study
P. pluvian	Paranaíta, MT	-/MN010726	D01	Voucher	This study
P. pluvian	Paranaíta, MT	-/MN010727	LA78	Voucher	This study
P. pluvian	Paranaíta, MT	-/MN010728	LA39	Voucher	This study
P. pluvian	Cotriguaçu, MT	-/MN010729	ABAM1555	Voucher	This study
P. pluvian	Cotriguaçu, MT	-/MN010730	ABAM3222	Voucher	This study
P. pluvian	Cotriguaçu, MT	-/MN010731	ABAM3364	Voucher	This study
P. pluvian	Cotriguaçu, MT	-/MN010732	ABAM3221	Voucher	This study
P. pluvian	Ilha Juruena, MT	-/MN010733	ABAM794	Voucher	This study
P. pluvian	Cotriguaçu, MT	-/MN010734	ABAM3349	Voucher	This study
P. latro	Altamira, PA	MK992578/-	BLM1010	Voucher	This study
P. latro	Altamira, PA	MK992579/MN010735	EA252	Voucher	This study

P. latro	Altamira, PA	MK992580/-	EA253	Voucher	This study
P. latro	Altamira, PA	MK992581/-	EA254	Voucher	This study
P. latro	Altamira, PA	MK992582/-	EA257	Voucher	This study
P. latro	Altamira, PA	MK992583/-	EA259	Voucher	This study
P. latro	Altamira, PA	MK992584/MK689168	LZA1335	Voucher	This study
P. latro	Altamira, PA	MK992585/-	EA262	Voucher	This study
P. latro	Altamira, PA	MK992586/-	EA255	Voucher	This study
P. latro	Altamira, PA	MK992587/-	EA256	Voucher	This study
P. latro	Anapu, PA	KX242519/-	LZATM 467	Voucher	This study
P. latro	Anapu, PA	KX925980/-	LZATM 743	Voucher	This study
P. latro	Anapu, PA	KX925981/-	LZATM 739	Voucher	This study
P. latro	Anapu, PA	KX925982/-	EH1388	Voucher	This study
P. latro	Anapu, PA	KX925983/-	LZATM 744	Voucher	This study
P. latro	Anapu, PA	KX242520/-	EH1416	Voucher	This study
P. latro	Santarém, PA	MK992590/-	UFOPA403	Voucher	This study
P. latro	Santarém, PA	MK992588/-	UFOPA404	Voucher	This study
P. latro	Santarém, PA	MK992589/MN010737	UFOPA413	Voucher	This study
P. latro	Anapu, PA	KX925986/MN010738	LZATM 751	Voucher	This study
P. latro	Altamira, PA	MK992591/-	EH1109	Voucher	This study
P. latro	Altamira, PA	MK992593/-	BLM1218	Voucher	This study
P. latro	Altamira, PA	MK992592/-	BLM1111	Voucher	This study
P. latro	Altamira, PA	MK992594/-	BLM1214	Voucher	This study
P. latro	Altamira, PA	KX242522/-	LZATM 277	Voucher	This study
P. latro	Altamira, PA	MK992595/-	EH1049	Voucher	This study
P. latro	Altamira, PA	MK992596/-	EH1051	Voucher	This study
P. latro	Altamira, PA	MK992597/-	BLM1085	Voucher	This study
P. latro	Altamira, PA	MK992598/-	LZA1351	Voucher	This study
P. latro	Altamira, PA	MK992599/-	LZA1352	Voucher	This study
P. latro	Altamira, PA	MK992600/-	EH1883	Voucher	This study
P. latro	Altamira, PA	MK992601/-	BLM1087	Voucher	This study
P. latro	Altamira, PA	-/MK689169	BLM 1086	Voucher	This study
P. latro	Senador José Porfírio, PA	MK992602/MN010736	EH1467	Voucher	This study
P. latro	Senador José Porfírio, PA	KX925985/-	LZA 748	Voucher	This study
P. latro	Medicilândia, PA	MK992603/-	EH968	Voucher	This study
P. latro	Medicilândia, PA	MK992604/-	EH980	Voucher	This study
P. latro	Medicilândia, PA	MK992605/MN010739	EH1003	Voucher	This study
P. latro	Medicilândia, PA	MK992606/-	EH996	Voucher	This study
P. latro	Medicilândia, PA	MK992608/-	CL12	Voucher	This study
P. latro	Medicilândia, PA	MK992607/-	CL13	Voucher	This study
P. latro	Medicilândia, PA	MK992609/-	EA133	Voucher	This study
P. latro	Medicilândia, PA	MK992610/-	EA134	Voucher	This study
P. latro	Altamira, PA	MK992611/-	LZA1336	Voucher	This study
P. latro	Brasil Novo, PA	MK992612/MN010740	EA251	Voucher	This study
P. latro	Medicilândia, PA	MK992613/-	EA998	Voucher	This study
P. latro	Volta Grande, PA	MK992614/-	EA260	Voucher	This study

P. latro	Volta Grande, PA	MK992615/-	EA261	Voucher	This study
P. latro	Senador José Porfírio, PA	MK992616/-	EH1468	Voucher	This study
P. latro	Senador José Porfírio, PA	MK992617/-	EH1520	Voucher	This study
P. latro	Altamira, PA	MK992618/-	BLM1136	Voucher	This study
P. latro	Altamira, PA	MK992619/MN010741	EA195	Voucher	This study
P. latro	Marabá, PA	-/KU494666	CFBHT12079	Voucher	This study
P. latro	Marabá, PA	-/KU494663	CFBHT12081	Voucher	This study
Pristimantis	Juruti, PA	MK992620/MN010742	MAP22	Voucher	This study
P. fenestratus	Borba, AM	KX242528/MN010744	INPA-H 34565	Voucher	De Oliveira et al. 2017
P. fenestratus	Borba, AM	KX242529/MN010745	INPA-H 34573	Voucher	De Oliveira et al. 2017
P. fenestratus	Borba, AM	KX242530/-	INPA-H 34571	Voucher	De Oliveira et al. 2017
P. fenestratus	Manaus, AM	MK992621/-	EA216	Voucher	This study
P. fenestratus	Manaus, AM	MK992622/-	EA214	Voucher	This study
P. fenestratus	Manaus, AM	/MN010746	EA217	Voucher	This study
P. fenestratus	Manaus, AM	MK992623/MN010747	EA218	Voucher	This study
P. fenestratus	Manaus, AM	MK992624/MN010748	EA219	Voucher	This study
P. fenestratus	Manaus, AM	KX242531/MN010749	EA210	Voucher	This study
P. fenestratus	Manaus, AM	MK992625/MN010750	EA212	Voucher	This study
P. fenestratus	Manaus, AM	MK992626/-	EA211	Voucher	This study
P. fenestratus	Manaus, AM	MK992627/MN010751	EA215	Voucher	This study
P. fenestratus	Manaus, AM	KX242533/-	EA213	Voucher	This study
P. fenestratus	Manaus, AM	KU495460/KU494667	CFBHT05715	Voucher	
P. fenestratus	Manaus, AM	KU495458/KU494665/	CFBHT05708	Voucher	
Pristimantis	Serra do Pardo, PA	MK992628/MN010743	SA37	Voucher	This study
P. fenestratus	Borba, AM	KX926012/MN010752	INPA-H 34580	Voucher	De Oliveira et al. 2017
P. fenestratus	Borba, AM	KX926013/-	INPA-H 34579	Voucher	De Oliveira et al. 2017
P. fenestratus	Borba, AM	KX926014/-	INPA-H 34577	Voucher	De Oliveira et al. 2017
P. fenestratus	Borba, AM	KX926015/MN010753	INPA-H 34562	Voucher	De Oliveira et al. 2017
P. fenestratus	Borba, AM	KX926016/-	INPA-H 34578	Voucher	De Oliveira et al. 2017
P. fenestratus	Borba, AM	KX926017/MN010754	INPA-H 34575	Voucher	De Oliveira et al. 2017
P. fenestratus	Peru	EU192277	MHNC3130	Voucher	
P. fenestratus	Bolivia	EU192276/-	MNCN 43031	Voucher	
P. fenestratus	Bolivia	EU192275/-	MNKA 6631	Voucher	
P. fenestratus	Bolivia	EU192274/-	MNKA 6630	Voucher	
P. samaipatae	Bolivia	EU192292/-	MNCN 42987	Voucher	

P. samaipatae	Bolivia	EU192289/-	MNKA 6626	Voucher	
P. chiastonotus	French Guiana	JN691300/-	183MC	Voucher	
P. chiastonotus	French Guiana	JN691303/-	1362BPN	Voucher	
P. chiastonotus	French Guiana	JN691311/-	1679BPN	Voucher	
P. koehleri	Bolivia	EU192282/-	MNCN 43013	Voucher	
P. koehleri	Bolivia	EU192279/-	MNKA 6627	Voucher	
P. zeuctotylus	Monte Alegre, PA	MK992629/ MK689171	RF29	Voucher	This study
P. zeuctotylus	Monte Alegre, PA	MK992630/MK689172	RF36	Voucher	This study
P. zeuctotylus	Monte Alegre, PA	MK992631/MK689173	RF74	Voucher	This study
P. zeuctotylus	Alenquer, PA	MK992635/-	MPEG29688	Voucher	This study
P. zeuctotylus	Óbidos, PA	MK992632/-	MPEG29437	Voucher	This study
P. zeuctotylus	Óbidos, PA	MK992633/-	MPEG30124	Voucher	This study
P. zeuctotylus	Óbidos, PA	MK992634/-	MPEG30892	Voucher	This study
P. achatinus	Colombia	JN371033/ JN371122	UVC:15953		
P. achatinus	Colombia	JN104676/ JN371121	UVC:15867		
P. conspicillatus	Ecuador	EF493529/	QCAZ28448		
P. skydmainos	Peru	EF493393			
P. vilarsi	Colombia	KP149384/ KP149187			
P. vilarsi	Colombia	KP149438/ KP149232			
P. bipunctatus	Peru	KY594758/ KY962785			
P. buccinator	Peru	EU712631	MNCN 9506		
P. carranguerorum	Colombia	KP149324/ KP149128	LSB 385		
P. crucifer	Ecuador	EU186718			
P. gaigeae	Panama	FJ784490/ FJ766788	KRL 1202		
P. lymani	Ecuador	EF493392	KU218019		
P. citriogaster	Ecuador	JQ964420	QCAZ:41487		
P. savagei	Colombia	KP149425/KP149220	LSB 381		
P. condor	Ecuador	EF493701	KU217857		
P. malkini	Ecuador	EU186663	QCAZ28296		
P. peruvianus	Peru	JN991461/ JN991392	AJC 2025		
P. terraebolivaris	Trinidad and Tobago	EU186650			
O. quixensis	Brazil	KU495404/ KU494611	MTR_ALCX186P53		
