# Supplementary material for: Four new species of Pristimantis Jiménez de la Espada, 1870 (Anura: Craugastoridae) in the eastern Amazon
Source: PLoS One. 2020 Mar 18;15(3):e0229971. doi: 10.1371/journal.pone.0229971 (PMC7080247; doi:10.1371/journal.pone.0229971)
Supplement: S3 File — (PDF) [file pone.0229971.s003.pdf]

Morphometric measurements of *Pristimantis* in this study.

Species	Locality	Exemplary	Sex	SVL	ThL	FL	HL	HW	IoD	EW	ID	DEN	EL	TL	TiL	HaL	SL	LL	TaL	AL
P. latro	Anapu, PA	LZATM 467	F	40	20.5	18.9	15.6	14.5	3.9	3.6	3.1	5.7	4.5	1.8	22.8	10.5	7.9	30.3	11.9	8.9
P. latro	Anapu, PA	EH1386	F	19.1	10	8.3	7.5	6.5	2.1	2	1.9	2.9	2.5	1	11.6	5.2	3.9	14.3	6.2	4.3
P. latro	Anapu, PA	EH1387	M	16.9	8.9	7.3	6.9	5.7	2	1.5	1.6	2.4	2.5	0.8	9.7	4.4	3.4	11.8	4.5	3.6
P. latro	Anapu, PA	EH1398	F	40.2	20.4	19	16.2	15	3.3	3	3.7	5.8	4.7	2.1	22.8	10.1	8	29.4	10.6	8.8
P. latro	Anapu, PA	EH1399	F	22.8	12.8	11	9.6	8.3	2.4	2.2	2	3.3	3.6	1.2	13.9	5.9	4.7	17.1	7	5.6
P. latro	Anapu, PA	EH1400	F	21.7	11.5	10	8.7	7.6	2	2.1	1.9	3	3.1	1.1	12.7	5.5	4.7	16.4	6.8	4.7
P. latro	Anapu, PA	EH1401	F	26.4	14.3	12.5	10.3	9.5	2.8	2.7	2.3	3.7	3.3	1.5	15.2	6.8	5.3	19.9	8.4	5.8
P. latro	Anapu, PA	EH1402	F	26.4	14.5	11.7	10.7	9.4	2.2	2.4	2.1	3.8	3.2	1.2	15.7	6.9	5.6	20.4	8.4	6.2
P. latro	Anapu, PA	EH1427	F	18.8	9.7	8.8	7.6	6.1	1.6	2.2	1.7	2.8	2.8	1	10.7	4.4	3.8	13.8	6.2	4.4
P. latro	Anapu, PA	EH1467	F	26.8	14.5	13.9	10.6	9.8	2.5	2.9	2.4	3.9	3.2	1.4	16.6	7.3	5.3	21.9	8.9	5.9
P. latro	Anapu, PA	EH1468	F	27.8	13.5	14.6	10.9	9.9	2.6	2.3	2.6	3.8	3.3	1.4	17.2	7.2	5.6	22.9	8.2	6.4
P. latro	Anapu, PA	EH1470	F	24.4	12.3	11.8	9.2	7.9	2.2	2.4	2.2	3.1	3.1	1.1	13.8	6.1	4.5	18	7.2	5.5
P. latro	Altamira, PA	LZATM63	M	28.3	14.8	14.8	11.6	10.1	2.7	2.9	2.6	4.1	3.9	1.6	16.8	7.6	5.8	22.9	8.4	6.9
P. latro	Altamira, PA	LZATM139	F	38.3	20.4	14.8	15	13.6	3.4	3.3	3	5.5	4.4	2	23.1	10	7.6	28.1	12	8.4
P. latro	Altamira, PA	LZATM155	F	28.2	14.1	13.4	10.4	8.4	1.9	3.2	2.3	3.9	3.2	1.4	16.1	7.2	5.4	21.3	8.9	6.5
P. latro	Altamira, PA	LZATM213	F	36.2	18.5	17	13.6	13.6	2.7	3.5	2.9	4.8	4	1.9	21.1	8.8	6.3	27.4	10.7	8.3
P. latro	Altamira, PA	LZATM265	F	26.2	13.9	13.9	10.5	9.4	2.7	2.2	2.2	3.9	3.8	1.4	16	7.2	5	21.4	7.8	6
P. latro	Altamira, PA	LZATM270	F	32.4	18	18.4	13	11.8	3	3.1	2.8	4.8	4	2	21.2	9.7	6.2	28.5	10	8.5
P. latro	Altamira, PA	LZATM277	F	29.7	15.4	13.9	11.7	10.6	2.7	2.9	2.5	4.3	3.5	1.7	16.3	6.8	6.1	21.8	8.5	6.4
P. latro	Altamira, PA	LZATM280	F	24.8	13.2	12.5	10.4	10	2.3	2.8	2.3	3.5	3.4	1.2	15.1	6.8	5	19.7	8	5.8
P. latro	Altamira, PA	LZATM281	F	26.2	14.3	12.8	10.3	9.4	2.8	2.6	2.3	3.6	3.3	1.3	15.7	6.6	5	20.4	8	6
P. latro	Altamira, PA	LZATM386	F	36.2	19.1	19.2	14.4	13.4	3.7	3.5	3.1	5.5	4.4	1.8	21.7	10.6	7.4	28.9	10.5	8.5
P. latro	Altamira, PA	EH 1564	F	27.6	14.1	13.8	11	9.8	2.9	2.3	2.4	3.7	4.1	1.6	16.4	7.5	5.1	21.3	8.1	6
P. latro	Altamira, PA	LZATM 1112	F	25.7	12.6	13.2	10.3	9.3	2.4	3.1	2.5	3.6	3.7	1.4	14.9	7.4	5.1	20	7.3	6.1

P. latro	Brasil Novo, PA	LZATM137	F	35.5	18.5	16.4	14	13.8	2.9	2.7	2.7	5.1	4	1.4	19.9	8.9	7	25.7	9.5	7.7
P. latro	Brasil Novo, PA	LZATM138	F	38.2	20.3	18	15.3	14.4	3.3	3.3	3.3	5.4	4.6	2.2	21.8	9.6	7.7	28.7	10.9	8.8
P. latro	Brasil Novo, PA	LZATM197	M	25	12.9	12.4	10	9.4	2.7	2.1	2.3	3.7	3.4	1.4	15.7	6.8	5.2	19.9	8.1	6.3
P. latro	Brasil Novo, PA	LZATM 802	F	35.6	18.9	19.4	13.7	12.1	3.6	3.4	3	4.8	4.7	1.8	21.4	9.5	6.9	29.1	10.9	8.1
P. latro	Brasil Novo, PA	LZATM 876	F	24.9	14.8	12.8	10.2	9.1	2.5	2.7	2.4	3.6	3.7	1.4	16	7.3	4.6	19.9	8	6
P. latro	Medicilandia, PA	LZATM140	M	30.1	14.9	16.4	11.9	10.7	2.5	2.9	2.7	4.3	3.5	1.4	18.8	8.5	6.2	24.8	8.9	7
P. latro	Medicilandia, PA	LZATM141	F	35.9	18.1	18.8	13.3	12.5	3.4	3.1	2.9	5	4.4	1.5	22.7	10.3	7.3	29.7	11.2	8.3
P. latro	Medicilandia, PA	LZATM188	F	37.3	18.8	19.2	14	13	3	3.2	2.7	5.1	4.5	1.9	22.4	9.1	6.6	30	11.4	8.7
P. latro	Medicilandia, PA	LZATM222	F	35.8	13.9	14	9.8	9.4	2.3	2.2	2.1	3.4	3.1	1.4	16	7	5	21.2	7.9	5.9
P. latro	Medicilandia, PA	LZATM229	F	22.2	11.4	11.2	8.8	8.9	2	2	2	3.1	2.8	1.2	14	5.9	4.4	18.4	7.5	5
P. latro	Medicilandia, PA	LZATM230	F	25.6	13.8	13	10.1	9.2	2.4	2.2	2.2	3.4	3.4	1.4	15.3	6.7	4.7	21.1	7.8	5.9
P. latro	Medicilandia, PA	LZATM236	M	25	13.5	13	10.2	9.1	2.8	2.3	2.1	3.7	3.3	1.5	15.8	7	4.9	20.5	7.4	6.2
P. latro	Medicilandia, PA	LZATM243	F	36	18.9	18.5	14.1	13.2	3.4	3.1	3.2	5.3	4	1.9	21.4	10.4	7.3	28.3	10.6	8.8
P. latro	Medicilandia, PA	LZATM255	F	35	18.6	18.4	13.3	12.8	3.7	2.9	2.7	5	3.9	1.7	21.4	9.3	6.8	28.3	10.4	8.3
P. latro	Medicilandia, PA	LZATM 818	M	25.7	13.2	13	9.8	8.9	2.3	2.6	2.1	3.6	3.9	1.8	15.3	6.8	5.1	20	7.5	6.4
P. latro	Medicilandia, PA	LZATM 814	F	38.7	17.9	17.5	13.6	12.6	3.1	3.4	3	5	4.7	1.9	20.3	9.4	7.1	26	10	8.1
P. latro	Medicilandia, PA	LZATM 816	M	26.5	12.6	13.1	10.4	9.5	2.4	2.7	2.3	3.7	3.9	1.7	15	7.1	5	20.2	7.9	6.5
P. latro	Medicilandia, PA	LZATM 815	M	27	13.4	13.6	10.8	10.1	2.6	2.6	2.4	3.7	3.9	1.7	15.6	7.3	5.3	20.2	7.6	6.3
P. latro	Uruará, PA	LZATM355	F	41	21.4	19.8	16.1	16.2	3.6	4	3	5.6	5	2.4	22.9	12	7.1	30.7	11.3	11.4
P. latro	Uruará, PA	LZATM356	M	25.8	13.3	12.6	9.9	9.6	2.4	2.1	2	3.5	3.8	1.2	16.1	7.1	4.9	21	7.9	6.3
P. latro	Uruará, PA	LZATM357	F	25.9	13.2	12.8	10.8	9.8	2.3	2.2	2.4	3.5	3.5	1.3	15.2	7	5.1	20.3	7.5	6.3
P. latro	Uruará, PA	LZATM358	F	34	17.9	16.2	12.5	12.2	3	3.5	2.8	4.8	4	1.7	20	9	6.1	25.9	9.8	8.2
P. latro	Uruará, PA	LZATM359	F	29.8	16.4	16	12.5	11.5	2.4	2.5	2.4	4.1	4	2.3	17.9	8.8	5.5	23.7	8.8	7.7
P. latro	Uruará, PA	LZATM360	F	36.9	18.4	19.5	15	13.9	3.3	3.8	2.9	5.5	4.2	1.7	21.9	9.9	7	30.4	11	8.8
P. latro	Senador José Porfírio, PA	EH1520	F	23.2	11.5	10.7	8.9	7.8	2.1	2	2.1	3.2	2.9	1.2	14.2	5.6	4.6	17.5	7.1	5
P. latro	Senador José Porfírio, PA	LZATM 1125	F	29.7	17.1	15.3	11.6	10.8	3.2	3	2.6	4.5	4.3	1.7	18.4	7.7	5.9	23.7	9.3	6.9

P. latro	Senador José Porfírio, PA	LZATM 1140	F	25	13.8	12.8	9.8	8.7	2.3	2.8	2.1	3.3	3.1	1.5	14.7	6.9	4.6	18.8	7.5	5.8
P. zeuctotylus	Monte Alegre, PA	RF029	F	37.7	20.2	17.4	14.3	14.7	4.2	3.8	3.3	5.1	4.7	2.5	20.8	10	7.2	26.5	10.6	9.2
P. zeuctotylus	Monte Alegre, PA	RF036	F	36	19.7	17.2	14	14	3.2	4	3.1	5.2	4.6	2.5	20.5	10	7.6	25.7	10.2	8.6
Pristimantis sp.	Andiroba, PA	INPA 10841	F	40.1	17.7	16.8	13.3	14.1	3.4	4	2.7	5	4.5	1.5	20	9	7.3	26.1	10.4	7.5
Pristimantis sp.	Andiroba, PA	INPA 10843	F	40	19.2	16.9	14.5	14.3	4	3.9	3	5.2	4.4	1.7	20	8.7	7.9	26	10.5	7.6
Pristimantis sp.	Andiroba, PA	INPA 10842	M	27.8	14.7	13.7	10.5	10.4	3.2	3.1	2.4	3.5	3.3	1.2	16.1	7.6	5.5	21.2	8.4	5.9
Pristimantis sp.	FLONA Trairão, PA	INPA 27694	F	20	11.2	9.9	8.1	7.5	2.3	2.8	2	2.5	2.9	1	11.9	5.6	4.1	16.2	6.3	4.2
Pristimantis sp.	FLONA Trairão, PA	INPA 27695	F	28.3	14.2	13.7	11.4	10.3	3.2	2.8	2.7	4	3.7	1.6	16.3	6.8	6.2	20.2	8.2	7
Pristimantis sp.	FLONA Trairão, PA	INPA 27696	F	28	12.7	13.3	11	10.8	2.9	3.2	2.6	4	3.6	1.7	15.8	7.1	5.6	21.5	8.7	6.4
Pristimantis sp.	FLONA Trairão, PA	INPA 27698	F	20.3	11	11.3	8.4	7.6	2.5	2.4	2.2	2.5	2.8	1	12.6	5.7	4.1	17.3	6	4.4
P. giorgii	Marabá, PA	MPEG 34840	M	29.23	16.24	15.9	10.6	11.3	2.5	2.9	2.4	3.8	3.5	1.8	17.8	8.3	5.4	24.4	8.9	6.6
P. giorgii	Marabá, PA	MPEG 34843	F	39.1	22.5	21.2	15.3	15.3	3.6	4.1	3.4	5	4.7	2.5	24.5	11.9	7.9	31	11.4	9.3
P. giorgii	Marabá, PA	MPEG 34847	F	37.9	21.7	20	14.3	14.7	3.6	3.3	3.4	5.1	4.2	2.5	23.7	10.7	7.8	31	10.5	8.9
P. giorgii	Marabá, PA	MPEG 34839	M	27.5	16.24	14.5	10.9	10.7	3	3	2.5	3.8	3.7	2	16.9	7.6	5.7	21.9	7.8	6.2
P. giorgii	Marabá, PA	MPEG 34842	F	37.4	21.3	20.4	14.1	14.7	2.9	4.1	3.4	5	4.5	2	22.8	11.5	7.4	30	10.8	8.3
P. giorgii	Marabá, PA	MPEG 34846	M	31.7	19.1	18.3	12.5	12.2	3	3.2	2.9	4.6	4.2	2.3	21.1	9.9	6.6	27.7	9.9	7.7
P. giorgii	Marabá, PA	MPEG 34841	M	27.3	16.24	15.1	10.8	10.9	2.6	2.7	2.6	3.9	3.8	2.1	18.1	8.3	5.5	23.1	8.5	6.4
P. giorgii	Marabá, PA	MPEG 34845	M	28	16.3	15.8	11.2	11.1	2.7	3.4	2.8	4	4.1	1.7	17.5	8.2	5.8	23.1	7.8	6.3
P. giorgii	Marabá, PA	MPEG 34844	F	11.6	10.5	9.7	8	7.4	2.1	2.5	2	2.9	3	1.2	11.9	5.5	4.1	15.7	5.3	4.1
P. fenestratus	Itaituba, PA	MPEG 33480	M	325.4	13.5	12.4	9.7	9.3	2.1	3.12	2.4	3.3	3.3	1.6	14.6	6.7	5	18.8	7.3	5.7
P. fenestratus	Itaituba, PA	MPEG 33477	M	23.9	12	12.6	9.3	8.4	2.7	3	2.1	3.1	3.3	1.7	14.5	6.1	4.7	19.5	7.3	5

P. fenestratus	Itaituba, PA	MPEG 33478	F	36.2	17.2	18.9	13.7	11.9	3.2	3.4	3.2	4.8	4.3	2.2	21.7	9.2	6.8	28	10.9	8.4
P. fenestratus	Itaituba, PA	MPEG 33479	M	26.2	13.3	12.9	10.2	9.3	2.4	3.2	2.5	3.4	3.8	1.8	15.3	7.2	5.1	19.6	7.5	6.2
P. "terra do meio"	ESEC Terra do Meio, PA	MPEG 29076	F	34.4	18.6	18.4	14.3	13.8	3.9	3.5	3.3	5.4	4	2.3	22.2	10	7.7	28.3	10.6	8.6
P. giorgii	Tucuruí, PA	MPEG 21146	F	37.2	19.8	19.2	14.3	13.4	3.5	3.3	3.4	5.3	4.5	2.5	23.5	10.7	7.6	29.2	12	8.5
P. giorgii	Tucuruí, PA	MPEG 21145	F	37	20.8	19.4	14.1	13	3.6	4.1	3.5	5	4.7	2.3	22.6	10.5	7.4	29.3	10.7	9
P. giorgii	Tucuruí, PA	MPEG 21147	M	25.2	13.2	12.8	9.9	9.2	2.8	3.3	2.4	3.4	3.5	1.4	15.1	7	5	19.8	7.1	6.8
P. giorgii	Canaã dos Carajas, PA	MPEG 35610	M	33	16.2	15.7	12	12	2.7	3.4	2.8	4.2	4.5	2.1	19.1	8.4	6.4	25.3	9.3	6.7
P. giorgii	Canaã dos Carajas, PA	MPEG 35613	F	41.2	22.2	19.5	15.2	15.9	3.3	4.3	3.5	5.2	5.1	2.3	23.5	10.6	7.5	30.2	11	9.2
P. giorgii	Canaã dos Carajas, PA	MPEG 35612	M	33	17.5	16.2	12.2	12.3	2.9	3.9	2.7	4.3	4	1.8	18.4	8.4	6.4	24.2	8.9	7.1
P. giorgii	Canaã dos Carajas, PA	MPEG 35611	M	32.8	15.3	14.1	11.9	11.5	2.8	3.3	2.9	4.2	4.3	2	16.8	7.4	6.2	21.9	7.9	6.3
P. giorgii	Canaã dos Carajas, PA	MPEG 16952	F	48.8	20.2	19.9	15	14.4	3	3.3	2.5	5.1	4.3	2.5	23.8	11.1	8.4	31.3	11.3	8.9
P. giorgii	Canaã dos Carajas, PA	MPEG 16960	F	40.7	20.9	20.7	15	15.1	3.4	4.2	3.3	5.4	5.1	2	23.7	10.5	7.8	30.9	11.2	8.8
P. giorgii	Canaã dos Carajas, PA	MPEG 16959	F	40.9	22.8	20.9	16.1	15.6	3.2	4.8	3.4	6	4.9	2.1	25.1	11.2	8.6	31.8	11.3	9.6
P. giorgii	Canaã dos Carajas, PA	MPEG 16958	F	41.6	22.9	22.1	16	16	3.7	4.6	3.9	6	5.6	2.2	26.5	12.1	9.1	32.1	12.1	9.4
P. giorgii	Canaã dos Carajas, PA	MPEG 16242	F	32.2	20	19.1	13.1	13.2	3	3.6	3	4.6	4.9	2	22	10.5	6.6	29.5	10.5	7.9
P. giorgii	Canaã dos Carajas, PA	MPEG 21221	F	37.8	21	19.7	13.4	14.4	2.7	4.7	2.7	4.6	4.7	2.2	22.3	10.3	7.2	28.5	10.5	8.2
P. giorgii	Canaã dos Carajas, PA	MPEG 21223	F	38.2	21.9	19.4	14.6	15.2	3.3	4.3	3.1	5.2	4.9	2.5	22.5	10.6	7.9	28.5	10.7	8.7
P. giorgii	Canaã dos Carajas, PA	MPEG 21226	F	27	14.6	12.8	10.7	10.3	2.6	2.8	2.2	3.6	3.5	1.6	15.4	7.1	5.7	20.4	7.2	5.5
P. giorgii	Canaã dos Carajas, PA	MPEG 21222	F	33.5	19.2	17.6	13.2	13.1	2.9	3.3	3	4.4	4.6	2.1	20.9	9.5	6.8	26.2	9.9	8

P. giorgii	Canaã dos Carajas, PA	MPEG 21225	F	35.4	21.1	19.3	13.7	14.6	3.3	4	3.2	4.8	4.8	2.3	22.3	10.2	7.2	28.3	10.6	8.4
P. giorgii	Canaã dos Carajas, PA	MPEG 17879	F	32.7	18.3	18	13.3	12.6	3.1	3.6	3.1	4.3	4.4	2.3	20.8	9.8	6.7	27.6	9.9	7.4
P. latro	Anapu, PA	MPEG 26050	F	41.6	21.7	19.4	16.1	15.8	3.7	4.4	3.7	5.7	5.8	2.5	24.5	11.3	8.2	29.8	11.4	8.9
P. latro	Anapu, PA	MPEG 26059	F	38	17.5	17.5	14.5	13.7	3.7	4.4	3.1	5.3	5	2.3	20.3	9.2	7.8	27	10	7.5
P. latro	Anapu, PA	MPEG 26052	M	27	12.1	12.8	9.8	9.2	2.6	2.6	2.3	3.6	3.6	1.4	14.5	7	5.1	19.3	7.3	6
P. latro	Anapu, PA	MPEG 26063	F	19.5	10.5	9.2	7.8	7	2.1	2.5	2.1	2.8	3.1	1	11.6	4.9	4	15.4	6	4.8
P. latro	Anapu, PA	MPEG 26065	F	19.5	10.7	9.4	7.8	7.4	2	2	1.7	2.6	3.1	1.2	11.4	5.2	4.1	13.9	6	4.3
P. latro	Altamira, PA	MPEG 31415	M	23.2	10.8	10.9	8.8	8.2	2.4	2.6	2.2	3	3	1.2	12.5	5.7	4.5	16.3	6.2	4.8
P. latro	Altamira, PA	MPEG 31416	M	26.2	12.2	12.8	10.1	9.8	2.7	3.1	2.3	3.4	4	1.6	14.8	6.7	5.1	19.5	7.5	5.5
P. latro	Altamira, PA	LZATM 1113	F	23.4	12.2	11.7	10	8.3	2.3	2.3	2.1	3.1	3.2	1.3	13.4	5.9	4.4	17.3	6.8	5
Pristimantis sp.	Santa Isabel do Pará, PA	MPEG 20602	F	25.5	15.3	13.5	10.6	9.8	2.5	3.4	2.6	3.5	3.7	1.6	16.9	7.9	5.4	21.9	7.8	6
Pristimantis sp.	Curionópolis, PA	MPEG 16859	F	37.5	22	20.3	13.3	14	3.4	3.6	2.7	4.6	4.4	2	24.3	11	6.6	31.4	11.6	8.9
Pristimantis sp.	Curionópolis, PA	MPEG 16879	J	21.5	11	10.6	8.5	7.7	2.1	2.4	2	2.9	2.9	1.3	13	6.1	4.3	16.6	6.4	4.3
Pristimantis sp.	Curionópolis, PA	MPEG 16917	F	29.8	15	14.3	12.2	11.9	2.9	3.1	2.6	4.3	3.9	1.9	17.4	8.6	6.2	20.9	8.5	6.8
Pristimantis sp.	Curionópolis, PA	MPEG 16918	F	38.2	20.8	20.7	15.2	14.8	3.4	4.2	3.3	5.2	4.7	2.2	23.6	11.2	8	30.4	11.6	8.6
Pristimantis sp.	Curionópolis, PA	MPEG 16926	F	37.5	21.6	20.3	14.2	13.9	3	4	3.2	5.1	4.8	2.2	23.3	11	7.3	30.1	10.9	8.8
Pristimantis sp.	Curionópolis, PA	MPEG 16899	F	32.6	20	18.9	13.6	13.2	2.8	3.8	3.1	4.3	4.6	2	21.3	10.7	6.9	28.6	11	7.5
Pristimantis sp.	Parauapebas, PA	MPEG 28456	F	41.2	21.3	20.4	14.8	15.5	3.5	4.5	3.4	5.3	5	2.3	24.9	11.1	8.1	30.6	12.6	9.2
Pristimantis sp.	Parauapebas, PA	MPEG 28458	M	29.7	15	14.4	11.3	11.6	2.8	3.5	2.6	4	4	1.8	16.9	8.1	6.1	22.6	8.3	6
Pristimantis sp.	Parauapebas, PA	MPEG 28454	M	31.5	15.7	16.5	11.7	12	2.8	3.4	2.7	4.1	4	1.7	18.8	8.1	6.1	24.9	9.1	6.9
Pristimantis sp.	Parauapebas, PA	MPEG 28457	F	41.8	20.7	21.1	15	15	3.4	4.5	3.4	5.1	4.5	2.8	23.4	11	7.6	30.1	11.2	8.6

Pristimantis sp.	Parauapebas, PA	MPEG 28455	M	30	13.8	14.3	10.9	11	2.4	2.9	2.6	4.1	3.8	1.9	16.8	8.2	5.9	22	8.2	5.9
Pristimantis sp.	Parauapebas, PA	MPEG 19886	F	36.3	18.2	17.9	13.4	12.9	3.1	4	3.2	5.2	4.8	2.1	21.5	9.4	7.4	27.5	10.9	8.1
Pristimantis sp.	Parauapebas, PA	MPEG 19887	F	37.6	19	17.8	14	13.2	3	3.5	2.9	5.2	4.6	2.2	21.8	9.9	7.3	26.3	10.8	8.9
Pristimantis sp.	Parauapebas, PA	MPEG 19853	F	34.9	17.2	17.2	12.8	12.6	2.6	3.6	2.9	4.8	4.5	1.8	19.7	9.1	6.9	25.4	9.9	7.6
Pristimantis sp.	Parauapebas, PA	MPEG 19889	M	30.2	15	14.9	11.4	11.2	2.6	3.7	2.7	3.9	4.4	1.6	17.3	8.3	6	22	8.5	6.2
Pristimantis sp.	Parauapebas, PA	MPEG 19888	F	42	21.5	20.3	15.1	15.3	3.5	4.9	3.5	5.2	5.2	2.2	24.5	10.9	7.4	31.1	11.9	9.6
Pristimantis sp.	Parauapebas, PA	MPEG 27664	F	39.3	21.7	20.6	15.3	14.9	3.1	4.2	3.4	5.1	4.7	2.3	23.6	11.2	7.9	30.4	12	8.8
Pristimantis sp.	Parauapebas, PA	MPEG 27667	M	23.9	14.6	13.1	9.2	9.6	2.5	2.9	2.1	3.4	3.4	1.5	15.6	6.9	4.7	18.5	7.4	6.1
Pristimantis sp.	Parauapebas, PA	MPEG 27661	F	21.9	12.7	11.6	8.5	8.5	2.2	2.2	1.9	3	3.2	1.2	14.4	6.1	4.5	18.2	7.3	5.3
Pristimantis sp.	Parauapebas, PA	MPEG 27670	F	29.7	17.1	16	11.5	11.6	2.5	3.2	2.6	4.1	4.3	1.7	18.4	8.4	5.7	23	8.6	7.1
Pristimantis sp.	Parauapebas, PA	MPEG 27658	F	38.4	22.5	20.4	14.6	14.5	3.1	3.7	3.3	5.6	4.9	2	23.7	10.7	7.6	29.9	11.5	9
Pristimantis sp.	Parauapebas, PA	MPEG 27669	F	29.7	17.4	16.6	11.6	11.8	2.9	3.4	2.6	4.2	3.9	1.9	18.6	8.5	5.9	24	9.3	6.6
Pristimantis sp.	Parauapebas, PA	MPEG 27672	F	28.9	15	14.8	11.9	11.9	2.5	3.3	2.4	4.1	3.8	1.9	18.8	8.3	6.2	23	9.8	6.6
Pristimantis sp.	Parauapebas, PA	MPEG 27665	F	39.1	22.8	20.2	14.9	15.5	3.2	3.8	2.8	5.4	4.9	2.2	24.3	10.6	7.4	28.6	11.3	9.3
Pristimantis sp.	Parauapebas, PA	MPEG 27673	F	38	22.7	19.9	15	15	3.1	3.9	3.1	5	4.6	2.1	23.5	11.2	7.4	30	11.3	9
Pristimantis sp.	Parauapebas, PA	MPEG 27662	F	37.1	21.7	20.4	14.3	13.3	3.4	3.7	3.2	5.1	4.7	2	24	11.3	7.6	29.3	11.6	8.8
Pristimantis sp.	Parauapebas, PA	MPEG 27674	F	30.1	17.4	16.1	11.9	11.8	2.8	3.4	2.8	4.1	4.4	1.8	18.7	8.5	6.3	24.5	8.9	6.8
Pristimantis sp.	Parauapebas, PA	MPEG 27666	F	37.6	22.9	20.4	14.5	15.5	3.2	4	3	5.3	4.6	2.1	24.1	10.6	7.4	29.9	11.8	9.7

Pristimantis sp.	Parauapebas, PA	MPEG 27668	F	38	20.8	19	14.2	15.3	3	4.2	3.1	5	4.8	2.6	22.3	9.4	7.1	28.4	10.9	8.2
Pristimantis sp.	Parauapebas, PA	MPEG 27659	F	37.3	20.9	19.7	14.6	14	3.4	3.9	3	5.3	4.6	1.9	22.1	10.6	7.7	28	11.3	8.6
Pristimantis sp.	Parauapebas, PA	MPEG 27663	F	36.2	21.4	19.3	14.3	13.9	3	3.5	2.9	5.2	4.4	1.9	23.3	11.1	7.5	29.1	11.6	8.5
Pristimantis sp.	Parauapebas, PA	MPEG 27663	F	36.3	21.1	19.5	14.3	13.8	2.9	3.5	2.9	5	4.8	2.3	23.3	11	7.3	29.9	11.6	8.3
Pristimantis sp.	Parauapebas, PA	MPEG 27660	F	30.3	15.5	13.7	12.1	11.5	2.6	3.4	2.8	4.1	4.5	1.8	15.9	7.7	5.9	18.8	7.6	6.5
Pristimantis sp.	Parauapebas, PA	MPEG 27671	F	27.8	15.4	14.5	10.8	10.5	2.2	3.2	2.4	3.7	3.8	1.7	16.3	8	5.7	21.8	8.5	6.3
Pristimantis sp.	Barcarena, PA	MPEG 14544	M	33.3	16.7	15.4	11.3	11.9	3	4	2.7	3.9	4.2	1.6	17.3	7.8	5.8	22.5	8.3	7.5
Pristimantis sp.	Barcarena, PA	MPEG14578	M	29.5	16.7	15.5	11.8	10.6	2.8	3.7	2.6	4.1	4.2	1.4	17.3	8.3	6.1	23.4	8.5	7
Pristimantis sp.	Barcarena, PA	MPEG 14579	F	40.5	21	21	15.3	15	3.6	4.5	3.6	5.6	4.9	2.2	23.3	11.3	8.2	30.7	11.5	9.2
Pristimantis sp.	Barcarena, PA	MPEG 14543	F	39.7	21.9	19.7	14.5	14.9	3.7	4.9	3.5	5.1	4.9	2	22.8	10.7	7.6	30.5	11.4	9.3
Pristimantis sp.	Barcarena, PA	MPEG 20252	F	37.3	20	19.6	13.7	13.2	3.3	3.8	3.3	4.9	4.9	2.1	22.7	10.2	7.4	30.4	12.1	8.9
Pristimantis sp.	Barcarena, PA	MPEG 20253	F	35.9	18.5	18.3	13.4	13.4	3.5	4.3	3.1	4.9	4.7	2.1	21.7	10.5	7.2	28.1	11.6	8.7
Pristimantis sp.	Barcarena, PA	MPEG 20261	F	26.7	14.1	13.5	10.6	10	2.6	2.8	2.6	3.4	4.3	1.6	16.2	7.9	5.6	21.2	8.1	6.4
Pristimantis sp.	Barcarena, PA	MPEG 20262	F	27.2	13.8	13.6	10.4	10.2	2.6	2.7	2.4	3.6	3.8	1.5	15.7	7.3	5.3	20.1	8	6
Pristimantis sp.	Ourilândia do Norte, MT	MPEG 2998	F	34.5	19.2	18.7	13.6	12.8	3.4	3.9	3	5	4.4	1.7	21.6	10	7.3	28.1	10.9	8.3
P. giorgii	Caxiuanã, PA	MPEG 16184	M	27.5	14.4	14	10.6	10	2.3	3.6	2.5	3.6	3.9	1.4	15.9	7.4	5.4	21.1	8.1	6.1
P. giorgii	Caxiuanã, PA	MPEG 15746	F	21.4	10.2	9.1	8.2	7.5	2.2	2.4	2	2.9	3.2	0.9	11.6	5.6	4.4	14.1	6.1	4.2
P. latro	Volta Grande, PA	BLM 1085	M	25.2	12.7	12.1	9.4	9	2.3	3	2.3	3.2	3.6	1.4	13.4	6.6	5	17.4	6.3	5.3
P. latro	Volta Grande, PA	BLM 1087	M	28.1	14.1	13.9	9.9	9.4	1.3	2	1.2	2.3	4.1	1.5	16.9	8.3	5.8	23	8.3	6.8
P. latro	Volta Grande, PA	LZATM 1883	M	25.4	11.5	12.7	10.5	8.7	2.5	3.2	2.4	3.4	3.7	1.5	14.2	6.7	5.2	18.5	7.2	5.5

P. latro	Volta Grande, PA	BLM 1086	M	28.5	14	13.9	10.9	9.7	2.4	3.1	2.4	3.7	4.2	1.7	15.5	7.6	5.8	21	7.4	5.7
P. latro	Volta Grande, PA	MPEG 26055	M	26.6	13.6	12.3	10.5	9.3	2.6	3.1	2.4	3.8	3.9	1.7	14.7	6.8	5.6	17.5	7.1	5.9
P. latro	Volta Grande, PA	MPEG 26053	F	40.5	20.4	19.1	14.7	13.4	3.2	3.9	3.3	5.4	4.9	2	22.4	10.1	7.7	28.6	11.9	8.8
P. latro	Volta Grande, PA	MPEG 26054	M	26.1	13.1	12.8	10.3	9.2	2.3	3.3	2.4	3.6	3.7	1.5	15.2	7.5	5.4	19.6	7.9	5.7
P. latro	Brasil Novo, PA	EA 251	M	24.5	13.3	11.1	9.8	9	2.8	2.7	2.4	3.1	3.6	1.6	14.1	6.7	4.9	18.5	7.3	5.7
P. giorgii	Assurini, PA	BLM 1089	F	20.9	10.5	9.6	7.8	7.3	1.9	2.2	1.9	2.6	2.6	0.9	11.4	5.3	3.9	14.3	5.6	4.4
P. giorgii	Assurini, PA	LZATM 1191	F	33.4	19.9	17.5	12.7	12.4	3.3	3	3	4.1	4	1.9	21.2	9.5	5.9	27.5	9.6	7.3
P. giorgii	Assurini, PA	LZATM 1190	F	35.6	22.3	20	14.2	13.7	3.5	3.6	2.4	4.6	4.4	1.9	23.6	11	6.5	30.4	11.5	8.8
P. giorgii	Assurini, PA	LZATM 1188	F	33.5	18.9	18.6	13.3	12.6	2.8	2.9	2.7	4.4	4.5	1.8	21.7	9.4	5.8	27.8	10.3	8.3
P. giorgii	Assurini, PA	LZA 1387 SF126	M	26.6	14.7	13.2	10.7	9.7	2.7	3	2.3	3.4	3.7	1.5	15.4	7.3	5.2	20.4	7.7	6.1
P. giorgii	Assurini, PA	LZATM 1197	F	33.2	19.9	18.5	13.4	12.7	3	3.6	3	4.9	4.4	2	21.9	9.7	6.6	27.6	10.4	8.4
P. latro	Altamira, PA	BIOTA 1214	M	28	14	12	10.7	9.7	2.5	3.5	2.4	3.6	3.9	1.5	14.8	6.3	5.3	19.7	7.7	6.4
P. latro	Altamira, PA	BIOTA 1102	F	38.2	20.1	18.6	14.9	14	3.1	3.9	3	5.1	4.6	2.2	22.1	9.6	7.3	28.6	11.4	8.3
P. latro	Altamira, PA	BIOTA 1103	F	28.5	15.5	14.2	11.4	10.5	2.7	3	2.4	4.1	3.8	1.8	16.5	7.1	6	21.7	9.1	7.1
P. latro	Altamira, PA	BIOTA 1218	F	40.9	19.3	19.6	15.2	14.5	3.5	3.6	3.2	5.6	4.7	1.9	21.9	10.1	7.9	28.8	11.7	8.8
P. latro	Altamira, PA	BIOTA 1111	F	40	20.8	20	15.3	14.6	3.5	4.1	3.9	5.9	4.5	2.3	22.1	10.2	8.2	28.9	11.1	9.5
P. fenestratus	Borba, AM	INPA 34571	M	32.8	16.4	17	12.8	12.3	2.3	3.9	2.5	4.3	4.3	1.8	17.8	9.3	6.5	24.6	8.5	7.7
P. fenestratus	Borba, AM	INPA 34577	M	30.8	15.3	14.5	11.3	10.7	2.4	3.6	2.9	3.8	4.5	1.5	16.8	7.6	5.6	22.1	8.6	6.4
P. fenestratus	Borba, AM	INPA 34562	F	32.4	15.7	15.4	11.9	11.1	2.6	4.1	2.7	3.9	4.4	1.8	16.2	7.8	6.5	21.6	7.2	6.7
P. fenestratus	Borba, AM	INPA 34565	M	31.4	15.1	14.1	11.9	10.3	2.8	3.6	3	4.4	4.4	1.7	16.7	8	6.1	21.6	8.5	6.2
P. fenestratus	Borba, AM	INPA 34573	M	34.3	19.1	17.8	12.6	11.6	2.6	4	2.9	4.5	3.9	1.6	19	10.2	6.5	26.2	9	7.7
P. fenestratus	Borba, AM	INPA 34580	M	31.1	14.4	14	11.6	10.2	2.6	3.8	2.8	4.2	4.3	1.6	16	7.4	6	21.1	8	5.7
P. fenestratus	Borba, AM	INPA 34579	M	30.7	15.3	14.6	11.7	10.8	2.6	3.9	2.9	4	3.8	1.7	16	7.8	5.8	21.9	8.8	6.5

P. fenestratus	Borba, AM	INPA 34578	M	28.6	15.4	15.9	10.4	10	2.3	3.5	2.5	3.8	3.4	1.3	17	9.1	5.6	23.8	8.8	6.1
P. fenestratus	Borba, AM	INPA 34575	M	31.6	15.9	16.3	11.1	10.5	2.3	3.3	2.6	4.2	4	1.4	17.5	8.7	5.7	23.3	8.2	6.8
P. fenestratus	Borba, AM	MPEG 7088	F	36	18.7	17.6	14	13.4	3.9	3.5	3.1	5.2	4.2	2.2	20.2	10	7.3	28.3	11.1	9.2
P. fenestratus	Desconhecida	MPEG 20594	F	32.3	16.5	17.3	12.5	11.6	2.8	3.4	2.6	4.7	4.2	2	19.9	9.1	6.6	26	9.7	7.4
P. fenestratus	Desconhecida	MPEG 20595	F	32.4	17.3	16.5	12.1	11.6	2.9	3.4	3	4.3	4.4	1.8	19.3	9.4	6.3	24.7	9.3	7.3
P. fenestratus	Desconhecida	MPEG 20593	F	31.2	16.5	16.5	11.9	11.5	2.9	3.2	2.6	4.5	4	1.7	19.7	8.8	6.5	24.1	10	7.2
P. fenestratus	Desconhecida	MPEG 20592	F	30.1	16.4	16	11.7	10.7	3.2	3.3	2.5	4.5	3.8	1.7	19.3	8.6	6.5	24.8	9.4	6.7
P. fenestratus	Desconhecida	MPEG 20591	F	33.6	17.4	16.8	12.4	11.8	3	3.7	2.7	4.7	4.3	1.9	20	9.1	6.4	26.2	10.4	7.5
P. fenestratus	Desconhecida	MPEG 20596	F	26.3	14	13.5	10	9	2.5	2.7	2.2	3.6	3.6	1.5	16	6.8	5.1	20.4	7.5	6.1
P. fenestratus	Desconhecida	MPEG 38567	F	24.1	13	13.1	10.1	9.9	2.3	3.1	2.5	3.6	3.4	1.2	15.9	7.2	5.1	20.5	8.1	5.8
P. latro	Santarém, PA	UFOPA 403	M	28	8.9	8.5	10.6	9.9	2.7	2.9	2.5	3.4	3.9	1.7	16.1	7.1	5.1	15.8	8.4	6.6
P. latro	Santarém, PA	UFOPA 404	M	30	16.4	15.3	11.2	10.8	2.6	2.8	2.4	3.4	3.8	1.8	17.3	8	5.2	23.2	9.2	6.7
P. latro	Santarém, PA	UFOPA 413	F	38.33	15.99	14.97	10.1	10	3.4	3.7	3.5	5.2	5.1	2.3	23.1	10.3	7.8	30.9	11.8	8.7
P. giorgii	Portel, PA	FUP 02	F	47.8	22.6	21.2	16.7	16.2	3.2	4.9	3.4	5.7	5.8	2.7	25.4	11.9	8.2	32.5	12.4	10.1
P. giorgii	Portel, PA	FUP 32	M	34.2	17.8	16.6	12.9	12.5	2.8	2.8	2.8	4.6	4.1	1.9	19.1	8.5	6.4	24.8	9.1	7
P. giorgii	Portel, PA	FUP 50	M	35	17.7	16.4	13.2	12.7	3	4	2.7	4.2	4.4	1.9	19.4	8.8	6.3	26	8.9	7.5
P. giorgii	Portel, PA	FUP 29	M	34.2	15.5	16	13.5	12.7	2.8	3.5	3	4.3	4	1.9	19.2	8.9	6.7	24.6	9.7	7.6
P. giorgii	Portel, PA	FUP 52	M	36.9	18.1	17.4	13.9	13	2.6	4	3	4.6	5	2.1	20.4	8.8	7	26.1	10.4	8.2
P. giorgii	Portel, PA	FUP 54	M	36.6	18.2	17.3	13.4	13.4	2.7	4.2	3.1	4.3	4.4	1.9	20.7	10.9	6.9	26.9	10.4	7.8
P. giorgii	Portel, PA	FUP 38	M	35.2	18.4	17.1	12.8	12	2.8	3	2.9	4.3	3.9	2	19.9	9	6.4	17.2	9.6	8.2
P. giorgii	Portel, PA	FUP 53	M	35.5	18	17.1	13.2	12.9	2.8	3.8	2.8	4.4	4.5	2.1	20.7	9.8	6.7	26.3	10	7.8
P. giorgii	Portel, PA	FUP 56	F	47	22.9	21.8	17.1	16.5	3.9	4.6	3.8	6	5	2.8	25.5	11.7	9.2	32.7	12	10.6

P. pluvian	Ipiranga do Norte, MT	ABAM 3040	F	41.1	22.6	20.5	16.2	14.1	3.3	3.7	3.2	5.5	4.7	2.3	23.5	10.8	8.2	30.2	11.1	8.4
P. pictus	Novo Mundo, MT	ABAM 2241	J	23.2	12.4	10.9	8.8	8	2.1	2.4	1.9	2.9	3	1	13.3	6.3	4.1	16.8	6.3	5.2
P. pluvian	Cotriguaçu, MT	ABAM 2173	F	40.48	21.3	18.6	15.9	14	2.9	3.2	3.1	5	4.1	2.4	23	9.6	7.7	28.3	11.1	8.4
P. pictus	Cotriguaçu, MT	ABAM 2146	F	38.6	20.9	19.8	15.3	15.1	3.2	3.4	3.3	5	4.8	2.2	23.4	11.6	7.7	30	11.6	10.2
P. pictus	Novo Mundo, MT	ABAM 2129	F	40.4	22.5	20.5	15.9	15	3.6	3.9	3.6	5.5	5	2.5	19.5	10.9	7.9	30.5	11.6	10.2
P. pictus	Novo Mundo, MT	ABAM 2155	F	43.6	22.3	20.8	16.5	14.8	3.7	3.7	3.3	5.2	4.9	2.5	24.2	10.4	8.2	30	10.9	9.6
P. pictus	Novo Mundo, MT	ABAM 2154	F	39	20.5	19.2	14.5	13.2	3	3.8	3	4.9	4.3	2.2	22.3	9.9	7.4	28.4	10.1	9.2
P. pluvian	Novo Mundo, MT	ABAM 2234	J	25.5	12.8	12.4	10	8.4	2.2	2.3	2	2.9	2.8	1.3	15.4	6	4.9	19.7	7.6	5.3
P. pictus	Cotriguaçu, MT	ABAM 2148	M	30.4	16	14	11.5	10	2.6	2.9	2.5	3.6	3.7	1.7	16.9	7.9	5.4	21.6	8.1	6.7
P. pictus	Novo Mundo, MT	ABAM 2109	F	38	21	19.9	14.3	14.2	2.9	3.5	3.1	5	4.1	2.1	22	10.5	7.7	29.6	10	9
P. pictus	Cláudia, MT	ABAM 2110	F	37.4	20	18.4	14	13.9	2.9	3.6	3.1	5.1	4.5	2.2	22	10	7.3	27.1	10.5	8.4
P. aff. *dundei*	Tabaporã, MT	ABAM 847	F	27.9	12.1	12.1	9.3	9.7	2.4	2.7	2.4	2.9	3.4	1.8	12.8	6.9	4.1	17.3	6.2	6
P. pictus	Caceres, MT	ABAM 2161	F	39.8	9.4	18.5	14.4	14.4	2.9	3.9	2.8	4.8	4.3	2.3	22.2	10.3	7	27.6	11	9.3
P. pictus	Novo Mundo, MT	ABAM 2150	F	33.8	16	14.8	12.1	11.4	3	3.3	2.7	4	4	1.6	17.1	8.4	6	21.4	8	7
P. pluvian	Novo Mundo, MT	ABAM 2233	J	26.2	12.6	11.8	9.4	8.3	2.4	2.2	2	2.9	3.4	1.2	15.4	6.4	4.6	19	7.4	5.6
P. pictus	Cotriguaçu, MT	ABAM 2151	M	30.6	15.6	15.2	11.7	11.3	2.6	3	2.4	3.9	3.9	1.6	18.1	8.8	5.8	23	8	6.2
P. pluvian	Cotriguaçu, MT	ABAM 394	M	32.5	15.7	15.2	11.8	10.5	2.4	3.5	2.6	3.7	4.6	1.5	17.9	8.2	6.1	23.5	8.3	6.4
P. pluvian	Cotriguaçu, MT	ABAM 843	F	32.2	18.5	17.3	13	12.2	2.7	3.5	3	4.1	4.6	2.3	20.2	8.3	6.6	26.1	9.2	7.1
P. latro	Belo Monte, PA	ABAM 3011	M	29.4	15.6	14.1	11.9	11.1	2.7	2.7	2.4	4.1	4.3	1.6	16.6	7.8	5.5	22	7.8	6.4
P. pluvian	Cotriguaçu, MT	ABAM 305	F	31.2	15.7	15.1	11.3	19.9	2.3	2.6	2.6	3.7	3	1.2	17.6	7.4	5.5	22.9	8.3	6.5
P. latro	Belo Monte, PA	BLM 1153	M	28	11.5	12.2	10.6	9	2.1	2.4	2.6	3.3	3.7	1.4	14.8	7	5.4	19.2	7.2	5.8
P. pluvian	Caceres (Mato Grosso)	ABAM 844	F	33.9	18.1	17.3	12.9	12.1	3	3.1	3	4.4	4.4	2.5	19.9	8.5	6.2	26	9.8	7.6
P. pluvian	Cotriguaçu, MT	ABAM 393	M	29.8	14.1	14.4	11.1	9.7	2.3	2.9	2.3	3.8	4.2	2.2	16.4	7.4	5.9	22.1	8.6	6.5
P. pluvian	Caceres, MT	ABAM 846	F	33.6	18.8	17.4	13.2	12.6	3.1	3.1	2.9	4.3	3.8	1.9	20.4	8.5	6.4	25.4	9.7	6.7
P. pluvian	Cotriguaçu, MT	ABAM 1556	F	41.7	20.4	19.8	15	13.6	3.2	3.8	3.5	5.4	4.4	2.2	23	9.8	8.3	29.4	11.2	8.5

P. pictus	Novo Mundo, MT	ABAM 1829	F	45.9	24.5	21.2	16.7	16.6	3.7	3.8	3.6	5.9	5.3	2.8	25.5	11.9	8.3	31	12.4	10.1
P. pluvian	Cotriguaçu, MT	ABAM 545	F	35.1	18.7	18.1	12.6	11.9	3.1	3.6	2.7	4.4	3.7	1.7	21.3	9.3	6.8	27.4	10.2	7.9
P. pictus	Novo Mundo, MT	ABAM 1602	F	43.6	22.5	20.3	15.8	15.3	2.9	4	3.3	5.2	5.3	2.2	24.3	11.5	7.7	29.4	11.8	9.8
P. pluvian	Cotriguaçu, MT	ABAM 1658	M	31	15.9	14.6	11.5	10.4	2.4	2.4	2.4	4	4.1	1.3	17.5	7.9	6.1	22.7	7.8	6.2
P. pluvian	Cotriguaçu, MT	ABAM 794	F	37.8	19.5	18.7	13.2	12.7	3.1	2.9	2.8	4.4	4.3	1.7	21.7	9.7	6.5	29	10.2	7.8
P. pluvian	Cotriguaçu, MT	ABAM 768	F	42	21.6	21.1	14.9	14	3.1	3.6	3.6	5.5	4.5	2.2	24.4	10.3	8.2	30.9	10.8	9.4
P. pictus	Cotriguaçu, MT	ABAM 1500	F	40.2	23	19.7	15.2	15.3	3.4	3.8	3.3	5.4	4.7	2.3	19.2	11.5	7.7	30	11.3	9.6
P. pluvian	Cotriguaçu, MT	ABAM 1555	F	43.1	21.1	20.9	14.6	13.8	3.2	3.2	3.3	5.1	4.5	2.3	23.4	10	7.8	31.1	12.1	8.6
P. pictus	Cotriguaçu, MT	ABAM 1525	F	30.3	15.7	14.1	10.8	10.8	2.6	2.8	2.5	3.8	4	1.6	17.8	8	5.5	22.3	8.4	7.2
P. pictus	Novo Mundo, MT	ABAM 1831	F	40.6	21.3	19	15	15.2	3.4	3.3	3.2	5.5	4.8	2.1	22.4	10.3	8	28.3	10.3	9
P. pluvian	Cotriguaçu, MT	ABAM 530	F	35.9	18.1	18	12.8	12.1	2.9	2.9	2.9	4.4	3.9	1.8	20.7	9	7	27	9.9	8
P. pluvian	Cotriguaçu, MT	ABAM 427	M	27.4	12.4	12.3	9.8	8.7	2.4	2.7	2.3	3.2	3.6	1.4	14.4	6.7	5.2	18.8	6.9	5.3
P. pluvian	Cotriguaçu, MT	ABAM 344	M	32	14.2	15.1	10.8	9.8	2.6	2.8	2.6	3.8	3.9	1.7	16.6	7.8	5.8	22.3	7.8	6
P. pluvian	Cotriguaçu, MT	ABAM 255	F	33	18.5	17.3	12	11.2	2.8	2.5	2.8	4.5	4	1.7	21	8	6.6	27.3	9.9	7.5
P. pluvian	Cotriguaçu, MT	ABAM 664	F	35.3	19.4	17.6	13.2	12.4	3	3.2	3.1	4.5	4.4	2.1	21.4	9.1	7	27.4	10.2	8.1
P. pictus	Novo Mundo, MT	ABAM 1830	F	40.5	22.8	18.6	14.6	14.8	3.3	4	3.1	4.9	5.3	2.2	23.5	11	7.7	29.5	10.3	9.2
P. pictus	Novo Mundo, MT	ABAM 1482	F	45.6	21.4	21.1	15.3	15.9	3.2	3.6	3.6	5.9	4.8	2.2	24.7	12	8.3	31.2	11.6	10.1
P. pluvian	Cotriguaçu, MT	ABAM 734	J	26.7	12.8	13.8	9.8	8.8	2.9	2.2	2.1	3.6	3.5	1.4	16.3	7.2	4.9	21	7.4	6.1
P. pluvian	Caceres, MT	ABAM 848	M	25.3	13	12.9	8.9	8.6	1.9	2.2	2	3	3.3	1.3	14.7	6.4	4.8	18.8	6.7	5.3
P. pluvian	Caceres, MT	ABAM 849	M	29.4	14.2	14	9.9	9.4	2.1	2.7	2.2	3.2	3.7	1.4	11.4	7.2	5.2	21.2	7.6	5.9
P. pluvian	Cotriguaçu, MT	ABAM 363	M	19.7	8.9	7.6	6	6.2	2.1	2.4	1.8	1.9	2.4	0.6	9.2	4.4	3	11.9	4.8	4.2
P. pluvian	Sinop, MT	ABAM 3078	M	28.1	14.9	13.6	10.4	9.6	2.6	2.5	2.1	3.3	3.2	1.5	15.8	8.2	5.2	20.5	7.7	5.5
P. pluvian	Sinop, MT	ABAM 3077	M	29.9	15.8	14.6	11.5	10.6	2.8	2.9	2.6	3.7	3.8	1.8	17.2	8.4	6	21.7	8.4	6.7
P. moa	Araguaina, TO	AMP 8550	F	39.2	21.9	20.9	15.5	15.3	2.9	4	3.3	5.4	4.7	2.2	23.5	9.9	7.9	31	10.7	9.3
P. moa	Palmeirante, TO	AMP 8557	F	40.6	21.5	21.7	15.6	14.4	3.1	4.4	3.3	5.6	4.9	2.2	23.8	12	8.3	32.5	11.3	9.4
P. pictus	Jacareacanga, PA	AMP 8540	M	29.9	15.5	15.2	12.2	11.5	2.6	3.5	2.8	3.7	3.9	1.5	17.5	8.1	6.1	22.6	7.7	6.1

P. pictus	Jacareacanga, PA	AMP 8539	M	28.7	14.2	13.8	11.5	10.8	2.6	3	2.3	3.7	3.8	1.8	16.6	7.8	5.6	21.2	7.7	6.4
P. pictus	Jacareacanga, PA	AMP 8538	M	28.9	14.9	13.4	11.1	11	2.3	2.8	2.2	3.6	3.8	1.6	15.5	7.8	5.7	20.1	6.9	6
P. moa	Araguaina, TO	AMP 8577	F	35.5	20.9	19.8	14.3	13.9	2.8	3.2	2.9	5.1	4.2	2	22.7	10.6	7.3	29.8	10.1	8.7
P. pictus	Jacareacanga, PA	AMP 8544	F	39.6	21.9	18.5	15.1	15.6	3.2	4.2	3.4	5.44	4.9	2.4	23.5	11.3	8	28.6	9.7	8.9
P. moa	Araguaina, TO	AMP 8548	F	40.6	22.6	20.6	15.3	15.7	3.3	4.4	3.2	5.7	5	2.6	23.8	11.3	7.9	31.9	11.8	9.6
P. moa	Araguaina, TO	AMP 8575	F	35.6	19.2	18.7	13.6	13.7	2.9	3.2	2.9	4.4	4.5	2.3	16.2	10.3	7	28.2	9.8	7.6
P. moa	Palmeirante, TO	AMP 8558	M	32.9	16.7	15.9	12.7	12.4	2.7	3.3	2.6	4	4.4	2.1	18.9	8.8	6.7	24	8.6	7.3
P. moa	Araguaina, TO	AMP 8576	F	43.4	21.4	20	16	15.9	3.3	4	3.5	5.2	5.1	2.4	22.4	11.5	8.6	30.7	11.3	8.3
P. pictus	Jacareacanga, PA	AMP 8541	F	38.9	20.4	19	14.7	14.7	3.1	3.7	3	4.8	4.8	2.2	22.4	10.7	7.8	28.4	10.2	8.6
P. pluvian	Cotriguaçu, MT	ABAM 3221	M	28.7	14.5	14	9.8	9.3	2.3	2.6	2.3	3.7	3.4	1.4	16.2	7.3	5.3	21.6	7.9	5.8
P. pluvian	Cotriguaçu, MT	ABAM 345	M	28.3	14.7	14.2	10.4	9.6	2.4	2.6	2.5	3.3	4	1.8	16.8	6.7	5.3	21.6	7.5	5.9
P. pluvian	Paranaíta, MT	AMP 8547	F	34.3	19.6	18	12.9	11.9	3	3.7	2.9	4.3	4.8	1.8	19.4	8.9	7	26.7	10	8
P. pluvian	Paranaíta, MT	AMP 8583	F	33	18.2	17.2	12.5	11.8	2.8	3.3	2.7	4.2	3.8	1.6	17.7	8.5	6.5	26.4	9.8	6.8
P. pluvian	Paranaíta, MT	AMP 8586	J	25.5	13.2	12.6	9.5	8.5	2.3	2.1	2.2	3.2	3	1.1	15.6	6.7	4.9	20.1	7.4	5.1
P. pluvian	Paranaíta, MT	AMP 8585	J	27.2	15.7	14.8	10.7	9.7	2.8	2.4	2.3	3.5	3.5	1.4	12.6	7.3	5.4	22.9	8.6	6.2
P. pluvian	Paranaíta, MT	AMP 8580	F	36	19.1	18.2	13	12.8	2.9	3.7	2.8	4.9	4.3	2.1	20.7	8.9	7.2	27.2	9.9	7.1
P. pluvian	Paranaíta, MT	AMP 8578	F	31.2	16.8	16.6	11.8	10.6	2.3	2.8	2.6	4.3	3.9	1.9	19.3	8.4	6.3	24.9	9.1	7
P. pluvian	Paranaíta, MT	AMP 8584	M	29.4	15.3	14.4	11.4	10.9	2.6	3.1	2.7	3.8	4.1	1.6	16.4	7.4	5.7	22.7	8.3	6.3
P. pluvian	Paranaíta, MT	AMP 8579	F	34.7	19.2	18.4	13.8	12.2	3	3.5	3.3	4.7	4.6	2	21.5	9.6	7.3	27.9	10.2	7.3
P. pluvian	Paranaíta, MT	AMP 8582	F	32.4	18	17.3	12.4	12	2.8	2.9	2.9	4.4	4.1	1.4	20.7	8.7	6.4	26.6	10	7.4
P. pluvian	Paranaíta, MT	AMP 8546	F	27.7	16	15.2	10.6	10.1	2.5	3.1	2.5	3.7	3.9	2	17.3	7.9	5.3	22.6	8	6.5
P. moa	Araguaina, TO	AMP 8574	J	24.3	13.1	12.1	9.9	9.4	2.3	2.7	2.2	3.4	3.3	1.7	14.9	6.9	5.1	19.2	7.7	5.7
P. moa	Araguaina, TO	AMP 8551	J	26.4	14.2	12.8	9.6	10	2.7	3.1	2.4	2.8	3.5	1.3	15.2	6.6	4.5	19.5	8	5.7
P. pictus	Jacareacanga, PA	AMP 8542	F	27.5	15.2	13.7	11.1	10.7	2.8	3.3	2.4	3.6	3.2	1.7	16.4	7.9	5.5	21.2	8	6.3
P. moa	Araguaina, TO	AMP 8549	F	30.4	16.5	15.4	11.7	11.8	3.1	3.1	2.6	4	4.2	1.8	17.3	7.9	6	22	8.7	6.6
P. pictus	Jacareacanga, PA	AMP 8543	M	25.7	14.6	13	9.9	9.2	2.7	2.9	2.4	3.3	3.7	1.6	15.6	7.3	5	20.1	7.2	6.3

P. pluvian	Cotriguaçu, MT	ABAM 549	F	41.1	21.2	20.9	14	13.2	3.6	3	3	4.6	4.5	2.2	23.6	10	7.1	31.4	11.3	9.2
P. pluvian	Cotriguaçu, MT	ABAM 739	F	30.1	14.9	15.3	11.4	10.1	3.1	2.6	2.6	3.8	3.5	1.9	17.5	7.9	5.6	21.9	8.6	6.3
P. pluvian	Cotriguaçu, MT	ABAM 770	J	24.1	12.4	12.4	8.7	8	2.1	2.1	1.9	2.9	3.1	1.2	14.3	6.7	4.3	19.1	6.7	5.2
P. pluvian	Cotriguaçu, MT	ABAM 735	J	24.9	12.9	12.6	9.5	8.7	2.5	2.2	2.1	2.9	3.4	1.8	15.6	7	4.8	19.6	7.8	6.1
P. pluvian	Cotriguaçu, MT	ABAM 550	J	29.2	14.2	14.5	10.8	9.5	2.4	2.5	2.2	3.4	3.9	1.5	16.9	7.3	5.3	21.6	8.1	6.1
P. pluvian	Cotriguaçu, MT	ABAM 740	J	27	14.2	14.8	10.2	9	1.8	2.5	2.2	3.3	3.5	1.7	16.2	7.3	5.1	21.6	7.6	6.6
P. pluvian	Cotriguaçu, MT	ABAM 781	J	23.5	12.8	12.4	9	8	2.4	2.2	2	3.1	3.3	1.7	14.3	6.4	4.3	19	7.7	5.2
P. pluvian	Cotriguaçu, MT	ABAM 741	J	29.1	14.5	14.2	10.4	9.9	2.7	2.9	2.3	3.4	3.6	1.9	16.6	7	5.2	21.1	8	6.5
P. pluvian	Cotriguaçu, MT	ABAM 742	M	29.8	14.8	14.7	11	10	2.7	3	2.7	3.8	4.1	1.9	16.3	7.6	6	22	8.7	6.6
P. pluvian	Cotriguaçu, MT	ABAM 531	F	37.5	21.1	19.9	14.3	12.7	3.1	3.4	3.4	4.8	4.3	2.2	23.6	10.5	7.7	24.9	11.1	8.4
Pristimantis sp.	Terra do Meio, PA	SA37	F	38.1	20.7	20.2	14.9	14.4	3.5	3.9	3.1	5.1	4.6	2.3	23	10.3	7.6	29.3	10.6	9.5
Pristimantis sp.	Terra do Meio, PA	JRSP6	F	37.7	20.4	18.5	15.3	14.1	3.5	3.4	3	5.1	4.8	2.2	22.3	10.4	7.4	27.7	10.6	9
P. moa	Riachão, MA	AMP 8587	M	34.4	17.1	16.2	12	13.1	2.9	3.2	2.8	4.3	4.6	2.3	18.3	9	6.3	23.3	8.6	7.6
P. moa	Riachão, MA	AMP 8591	M	31.2	16.5	14.6	12	12.2	2.5	3.3	2.7	4.3	4.6	2.2	16.5	8.2	6.5	22	7.9	7.1
P. moa	Riachão, MA	AMP 8589	M	33.3	16.8	16.4	12.7	13.3	2.7	3.4	2.5	4.5	5	2.1	18.2	9	6.4	24.4	8.4	7.4
P. moa	Riachão, MA	AMP 8593	M	31.8	15.8	14.6	11.3	12	2.4	3.3	2.6	4	4	2.1	16.6	8.3	5.9	22.3	7.7	6.8
P. moa	Riachão, MA	AMP 8590	M	31.3	15.4	14.6	11.8	12	3	3.4	2.7	3.9	4.1	2.2	16.1	8.3	5.8	21.1	7.7	6.4
P. moa	Riachão, MA	AMP 8592	M	30.6	15.8	15.1	11.2	11.7	3.1	3.3	2.8	4	4.3	2	16.6	8.6	5.6	22.6	7.8	7
P. moa	Riachão, MA	AMP 8588	M	31.3	16	15.1	11.8	11.9	2.8	2.9	2.6	4.1	2.2	2	16.7	8.7	6.4	22.5	8.2	7.2
P. moa	Riachão, MA	AMP 8594	M	32	16.1	15.9	12.3	12.7	3.1	3.1	2.6	4.3	4.6	1.8	17.3	8.8	6.2	23.7	8.5	7.6
